# Following the Pathway of W Chromosome Differentiation in *Triportheus* (Teleostei: Characiformes)

**DOI:** 10.3390/biology12081114

**Published:** 2023-08-10

**Authors:** Mariannah Pravatti Barcellos de Oliveira, Rafael Kretschmer, Geize Aparecida Deon, Gustavo Akira Toma, Tariq Ezaz, Caio Augusto Gomes Goes, Fábio Porto-Foresti, Thomas Liehr, Ricardo Utsunomia, Marcelo de Bello Cioffi

**Affiliations:** 1 Departamento de Genética e Evolução, Universidade Federal de São Carlos, Sao Carlos 13565-905, Brazil; mariannah.bo@gmail.com (M.P.B.d.O.); geizedeon@hotmail.com (G.A.D.); gustavo_toma@hotmail.com (G.A.T.); mbcioffi@ufscar.br (M.d.B.C.); 2 Departamento de Ecologia, Zoologia e Genética, Instituto de Biologia, Universidade Federal de Pelotas, Pelotas 96010-610, Brazil; rafael.kretschmer@ufpel.edu.br; 3 Faculty of Science and Technology, Centre for Conservation Ecology and Genomics, University of Canberra, Canberra 2617, Australia; tariq.ezaz@canberra.edu.au; 4 Faculdade de Ciências, Universidade Estadual Paulista, Bauru 13506-900, Brazil; caio.goes@unesp.br (C.A.G.G.); fp.foresti@unesp.br (F.P.-F.); ricardo.utsunomia@unesp.br (R.U.); 5 Institute of Human Genetics, University Hospital Jena, 07747 Jena, Germany

**Keywords:** cytogenomics, sex chromosomes, satellite DNA, FISH

## Abstract

**Simple Summary:**

The evolutionary origins and dynamics of sex chromosomes are among the most challenging topics in the field of Evolutionary Biology. Despite ongoing research, many important aspects of sex chromosome evolution remain unresolved. One intriguing question is why do sex chromosomes tend to accumulate species-specific repetitive sequences. In our current study, we delved into this issue by examining the variation in satellite DNAs (SatDNAs) during the W differentiation process in many *Triportheus* fish species. Our findings added valuable insights to this complex puzzle. Despite sharing a similar ancestry, the DNA composition of the sex chromosomes of *Triportheus* species differed significantly. Notably, the W chromosome evolved independently among its various species.

**Abstract:**

In this work, we trace the dynamics of satellite DNAs (SatDNAs) accumulation and elimination along the pathway of W chromosome differentiation using the well-known *Triportheus* fish model. *Triportheus* stands out due to a conserved ZZ/ZW sex chromosome system present in all examined species. While the Z chromosome is conserved in all species, the W chromosome is invariably smaller and exhibits differences in size and morphology. The presumed ancestral W chromosome is comparable to that of *T. auritus*, and contains 19 different SatDNA families. Here, by examining five additional *Triportheus* species, we showed that the majority of these repetitive sequences were eliminated as speciation was taking place. The W chromosomes continued degeneration, while the Z chromosomes of some species began to accumulate some TauSatDNAs. Additional species-specific SatDNAs that made up the heterochromatic region of both Z and W chromosomes were most likely amplified in each species. Therefore, the W chromosomes of the various *Triportheus* species have undergone significant evolutionary changes in a short period of time (15–25 Myr) after their divergence.

## 1. Introduction

Sex chromosomes represent important and dynamic components of the genome [1,2,3,4]. They are characterized by specific morphology, genomic content, and evolutionary mechanisms [2]. In general, they arise from an autosomal pair that acquires a putative sex-determining gene linked to DNA sequences that play sexual antagonistic roles [3,4,5]. This step is important to lead to partial or complete recombination suppression between the homologs [6]. In a recent investigation, [7] highlighted some neglected questions that would require a fine-tuning or partial remodeling of this canonical paradigm, emphasizing the need for a new perspective of sex chromosome evolution. Among vertebrates, although mammals and birds contain, respectively, stable XX/XY and ZZ/ZW sex chromosome systems in most species, in the majority of reptiles, frogs, and fishes, no distinct sex chromosomes can be found [8]. In fishes, although only 6% of fish species studied so far exhibit heteromorphic sex chromosomes, its species show both female (ZZ/ZW) or male (XX/XY) heterogamety and almost all their multiple derived forms [9]. A ZZ/ZW sex chromosomal system is present in roughly 115 species, with the Triportheidae family standing out among all reported cases [9]. Triportheidae is a monophyletic fish family composed of five genera: *Lignobrycon*, *Engraulisoma*, *Clupeacharax*, *Agoniates*, and *Triportheus* [10]. According to Yano et al. [11], the ZW sex chromosome system, shared by all Triportheidae species and some Gasteropelecidae members, had a common origin and most likely evolved from the last common ancestor of these two lineages. Therefore, the ZW sex chromosome system should be at least 40 My old. The genus *Triportheus* (the focus of this work) is the most specious in the family, currently comprising 21 species [12], standing out by their conserved ZZ/ZW sex chromosome system present in all analyzed species [11,13,14]. Their Z chromosome is metacentric and is the largest in the karyotype, but the W chromosome is usually smaller and varies in size and form [13,15,16,17,18,19]. Additionally, the W chromosome of all species is rich in heterochromatin and carries an 18S rDNA site specifically on the long arms [17,20,21,22]. Previous studies analyzed the relationship between W chromosome differentiation and the accumulation of repetitive DNA sequences by mapping different microsatellite motifs in various species. The results showed a preferential (but divergent among species) accumulation of repetitive sequences on the W chromosome [19,22,23]. 

With the development of Next-Generation Sequencing (NGS) technology, researchers have only recently started to delve deeply into the repetitive DNA found in these chromosomes and their impact on the mechanisms that lead to sex chromosome differentiation [24,25]. The satellitome, which is made up of the whole collection of satellite DNAs (SatDNAs) in a genome, offers an excellent opportunity to elucidate the dynamics of such components in the formation and upkeep of heteromorphic sex chromosomes [26,27]. Recent data provided a significant source of information for characterizing the satellitome of a variety of organisms, from insects [28,29,30,31,32] to fishes [25,33,34,35,36,37] frogs [38,39], lizards [40], birds [41], and mammals [42,43,44,45]. In general, SatDNAs are typically found in the centromeric, telomeric, and heterochromatic regions [46]. In general, the male (Y) or female (W) specific chromosomes highly accumulate SatDNAs, which results in significant disparities in sizes and genetic composition between the sex pair [32,34,37,47]

In a recent analysis, Kretschmer et al. [37] characterized and mapped the satellitome of *T*. *auritus*, integrating genomic and chromosomal data with a particular focus on the SatDNAs accumulating on the ZW chromosomes. The *T*. *auritus’* satellitome had 53 SatDNA families (named TauSatDNAs), 24 of which were also mapped by FISH in three *Triportheus* species (*T*. *auritus*, *T*. *albus*, and *T*. *signatus*) and in the sister group *Agoniates halecinus*. Most TauSatDNAs differed significantly between the sexes, with 19 and 3 of them being accumulated in the *T. auritus*’ W and Z chromosomes, respectively. However, there were only six and four SatDNAs found in the W chromosomes of *T. signatus* and *T. albus,* respectively [37]. For all TauSatDNAs hybridized on the chromosomes of *A*. *halecinus,* no FISH signal was observed. As a result, it is clear that there was a significant dynamic involved in the differentiation of the sex-specific chromosome (W) in relation to this major class of repetitive DNA.

Here, to investigate the dynamics of SatDNAs during the path of ZW sex chromosomes differentiation in a larger phylogenetic context, we mapped the 19 TauSatDNA families accumulated in the W chromosome of *T. auritus* (TAU) in five additional *Triportheus* species, covering almost all species (except *T*. *venezuelensis*) with available chromosomal data up to date. The results evidenced both the amplification and elimination of SatDNA repeats during the pathway of W chromosome differentiation, without a direct relationship of ancestry. 

## 2. Material and Methods

### 2.1. Samples and Chromosomal Preparations

Five *Triportheus* species, including *T. guentheri* (TGU), *T. nematurus* (TNE), *T. pantanensis* (TPA), *T.* aff. *rotundatus* (TRO), and *T. trifurcatus* (TTR) were examined (Table 1). Except for TPA, where only female chromosomal preparations were available, both male and female specimens of all species were examined. The experiments complied with ethical standards set by the Federal University of São Carlos Ethics Committee on Animal Experimentation (Process number CEUA 7994170423). Mitotic chromosomes were acquired using cells from the anterior portion of the kidney, as described in Moreira-Filho et al. [48]. Briefly, the animals were treated with a colchicine solution for 40–50 min. After this period, the kidney fragments were transferred to a hypotonic solution, dissociated, and incubated at 37 °C for 20 min. The cell suspension was fixed with Carnoy’s solution (3:1 methanol/glacial acetic acid) and dropped onto cleaned microscope slides.

### 2.2. Probe Labeling and Fluorescence In Situ Hybridization (FISH)

We selected the 19 TauSatDNAs (GenBank access number OL351494-OL351546) that were accumulated on the W chromosome of TAU previously described by Kretschmer et al. [37]. We labeled them using the Nick translation mix kit (Jena Bioscience, Jena, Germany) with Atto550-dUTP (red) or Atto488-dUTP (green) following the manufacturer’s manual. After that, target satellite probes were hybridized in the investigated species following the protocol described by Pinkel et al. [49]. Briefly, the slides containing metaphasic chromosome spreads were initially treated with RNAse solution for 1 h at 37 °C and pepsin solution (0.005%) for 10 min at 37 °C. Then, the slides were denatured in 70% formamide solution at 72 °C for 3 min. The probe mixture composed of 50% formamide, 2× SSC, dextran sulfate, and the target labeled probe, was denatured for 10 min and applied onto denatured chromosome slides. Hybridization was performed for at least 16 h in a moist chamber at 37 °C. After this period, post-hybridization washes consisted of 1× SSC at 65 °C, 4× SSC/Tween solution, and 1× phosphate-buffered saline at room temperature, respectively. The slides were dehydrated in an ethanol series, and after complete drying, the chromosomes were counterstained with DAPI/Antifading (Vector Laboratories, Newar, CA, USA).

### 2.3. Microscopy Analysis and Image Processing

In order to corroborate the diploid count and FISH data, we evaluated at least 30 metaphase spreads per species, which were consistent and exhibited the same results in all experiments. The photos were captured using the CoolSNAP system software, Image Pro Plus, 4.1 (Media Cybernetics, Silver Spring, MD, USA), and the Olympus BX50 microscope (Olympus Corporation, Ishikawa, Japan). Chromosomes were classified as acrocentric (a), metacentric (m), and submetacentric (sm), according to their arm ratios [50].

### 2.4. Polymerase Chain Reaction (PCR) Using the DOP-PCR Amplified Microdissected Z Chromosomes

Using the microdissected Z chromosomes previously obtained by Yano et al. [22] as a template, we conducted PCR reactions with all 19 TauSatDNA primers in order to also verify their presence in the Z chromosomes. The following amplification conditions were used: initial denaturation during 5 min at 95 °C, 30 cycles at 95 °C during 5 min, variable annealing temperature (39.1–54.2 °C) during 40 s, and 72 °C during 30 s, and a final extension step of 10 min. Details of the PCR conditions for the amplification of each SatDNA can be found in Kretschmer et al. [37] (Supplementary Table S1). The resulting PCR products were checked in a 2% agarose gel. 

## 3. Results

### 3.1. Chromosomal Location of TauSatDNAs 

**Figure 1:**
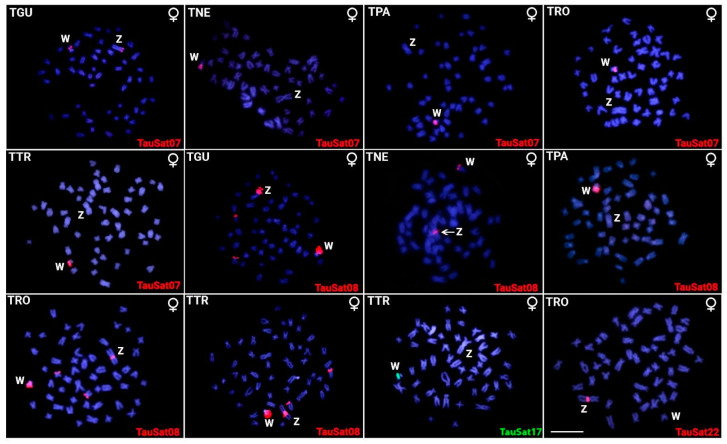
Female metaphase plates of *Triportheus guentheri* (TGU), *T. nematurus* (TNE), *T. pantanensis* (TPA), *T. rotundatus* (TRO), and *T. trifurcatus* (TTR) highlighting the chromosomal location of TauSatDNAs. While TauSat07 and TauSat08 showed positive hybridization signals in all species, TauSat17 and TauSat22 were exclusively mapped in TTR and TRO. The SatDNA family names are indicated on the right bottom, in green (Atto488 labeled) or red (Atto550 labeled). The ZW sex chromosomes are indicated. Bar = 5 μm.

**Figure 2:**
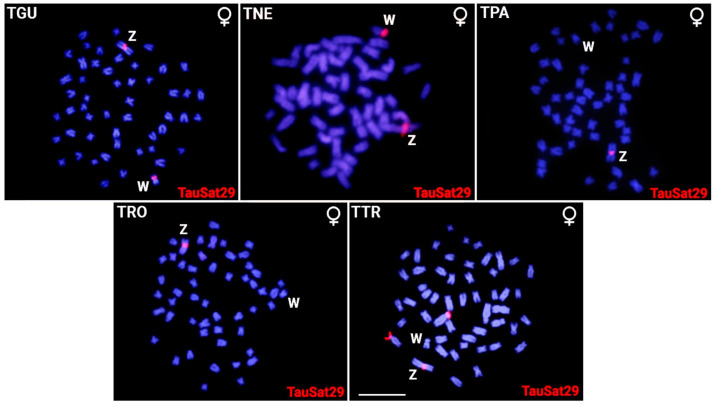
Female metaphase plates of *Triportheus guentheri* (TGU), *T. nematurus* (TNE), *T. pantanensis* (TPA), *T.* aff. *rotundatus* (TRO), and *T. trifurcatus* (TTR) highlighting the chromosomal location of TauSat29 in red (Atto550 labeled). The ZW sex chromosomes are indicated. Bar = 5 μm.

**Figure 3:**
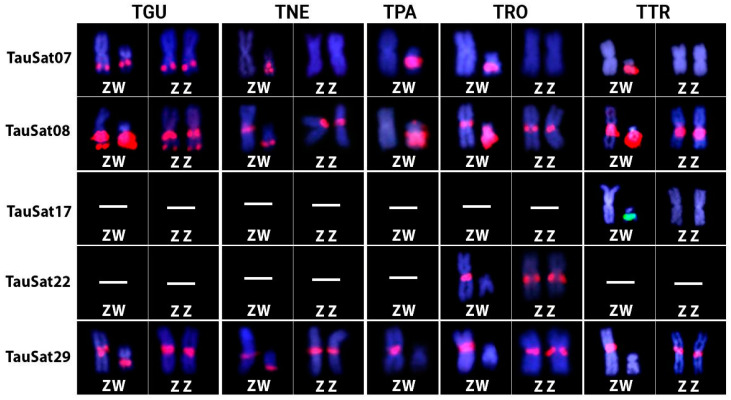
Detailed comparison of different TauSatDNAs that hybridized on the Z and/or W chromosomes of *Triportheus guentheri* (TGU), *T. nematurus* (TNE), *T. pantanensis* (TPA), *Triportheus* aff. *rotundatus* (TRO), and *T. trifurcatus* (TTR). The ZW chromosomes were extracted from Figure 1, Figure 2 and Figure S1.

Only 5 of the 19 SatDNAs mapped on the W chromosome of TAU [37] were found to be present in the Z and/or W chromosomes of the herein investigated *Triportheus* species. Among them, TauSat07 and TauSat08 were clustered in the W chromosomes of all species, with the latter being accumulated the most, taking up the entire Wq in the TGU, TPA, TRO, and TTR (Figure 1, Figure 2 and Figure 3). Contrarily, TauSat17 and TauSat29 were restricted to the W chromosomes of TTR, TGU, and TNE, respectively (Figure 1, Figure 2 and Figure 3).

Some SatDNAs were shown to be located on the Z chromosome. The Z chromosomes of all the analyzed species exhibited FISH signals for TauSat29, except for TPA and TauSat08. On the other hand, TauSat07 and TauSat22 were only mapped on the Z chromosomes of TGU and TRO, respectively. Except for TauSat07, which mapped the interstitially on Zq, they were all centromeric (Figure 1, Figure 2 and Figure 3). 

### 3.2. SatDNA Content of TAU Z Chromosome

All 19 TauSatDNAs that were exclusively mapped using FISH on the TAU W chromosome were amplified in the microdissected Z chromosomes, demonstrating that this chromosome also contains these SatDNAs, although probably in lower copy numbers, impairing their FISH location. Agarose gel images of PCR amplifications can be found in Supplementary Figure S2.

## 4. Discussion

In this work, we reconstruct the likely evolutionary pathways that led to the SatDNA patterns currently observed, which took place after the diversification of several Triportheus species. The combined findings from previous studies [19,22,37,51,52] and those from the present research further show the plasticity that affects the evolutionary history of *Triportheus* ZW sex chromosomes and the remarkably dynamic differentiation process of the female-specific W chromosome.

### 4.1. The W Chromosome Differentiation

**Figure 4:**
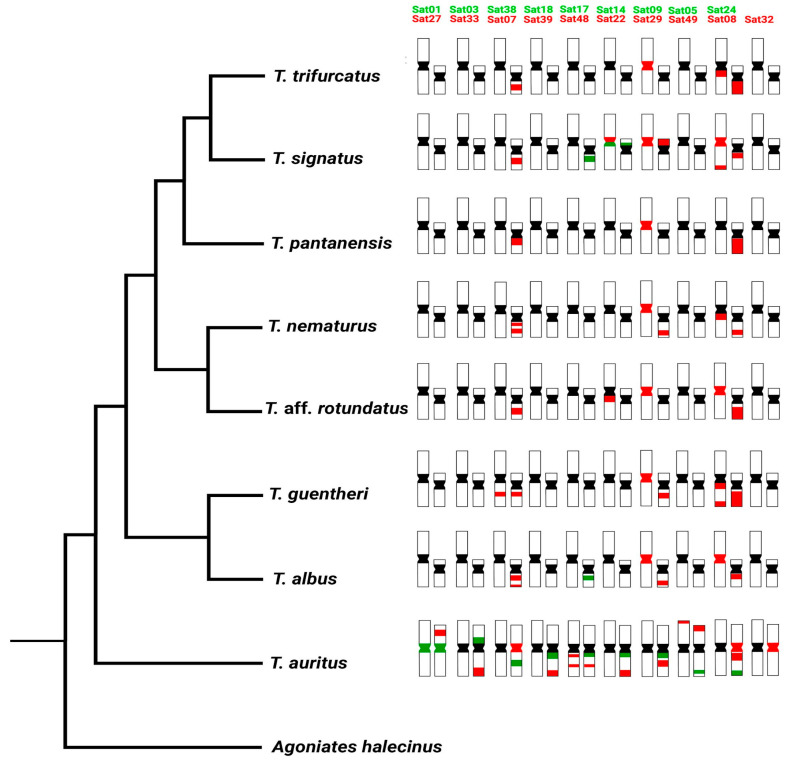
Phylogenetic relationships among the analyzed *Triportheus* species based on Mariguela et al. [10] and Melo et al. [53] with the representative ideograms highlighting the distribution of the TauSatDNAs on the Z (on the left) and W (on the right) chromosomes of *Triportheus*. Some data came from Kretschmer et al. [37].

To date, all *Triportheus* species exhibit a highly differentiated ZW sex chromosome system [11,19]. Despite the overall chromosomal similarities, our cross-hybridization experiments using TAU SatDNAs revealed distinct accumulation patterns among the analyzed W chromosomes. The largest W chromosome among all *Triportheus* species, with a similar size and shape to the Z, is found in TAU (sister species of all *Triportheus* that diverged approximately 20.7 ± 6.5 Myr) [10,22]. In this species, 19 TauSatDNA families are accumulated on the W, predominantly on its heterochromatic long arms [37], which are also home to a major 18S rDNA cistron [13]. From these, only two of them (TauSat07 and TauSat08) were ubiquitously accumulated in the W chromosome of all the other *Triportheus* species (Figure 4).

Recently, Goes et al. [54] characterized the satellitomes of the characiform fishes *Colossoma macropomum* and *Piaractus mesopotamicus*, and four TauSatDNAs were detected (TauSat06-42, TauSat19-76, TauSat16-29, and TauSat12-66) in these species; however, none of them were located on the sex chromosomes of any *Triportheus* species ([37], present data). This assertion is supported by the fact that despite having a ZW sex system, like *C. gomesi* [35] and *M. macrocephalus* [34], our analyses do not show any similarities between the SatDNAs found in the sex chromosomes of Triportheidae and other satellitomes of Characiforms. Therefore, the SatDNAs implicated in the evolution of their sex chromosomes are either species-specific or exclusively shared only by phylogenetically closely related species. As ZOO-FISH and CGH experiments demonstrated the correlation of the repetitive portion of sex chromosomes of Triportheidae and Gasteropelecidae [11] and given the close evolutionary relationship of these two families [53], future studies can examine the association between the SatDNAs accumulated in Triportheidae and Gasteropelecidae sex chromosomes. 

### 4.2. From W to Z Chromosomes

The evolution of a sex chromosome is assumed to take place via a progressive stopping of recombination between homologous autosomes [4]. Following that, gene degradation and repetitive DNA accumulation occur [55,56]. However, the dynamic changes in repetitive DNA content that affects the Z (or X) and the W (or Y) chromosomes are completely different [56]. Here, only 3 (TauSat01 (which makes up the centromere of all chromosomes of TAU), TauSat48, and TauSat49) out of the 19 TauSatDNAs investigated exhibit a detectable FISH signal on the Z chromosomes of TAU ([37], Figure 4). Interestingly, as speciation processes take place, the Z chromosomes of some species began to accumulate some TauSatDNAs while the W chromosomes lost most of the TauSatDNA repeats (Figure 4). Although unusual in various animal species, including fishes ([23] and in our work) and birds [57], repetitive sequence accumulation on the Z chromosome has been observed. In fact, these frameworks of SatDNA homogenization may act as a possible dosage mechanism, compensating for the losses of such sequences in one of the sex chromosomes, and maintaining the subdued recombination between the sex-determining and autosome regions, as the recombination loop may still form [58]. On the other hand, when SatDNAs accumulate in both the Z and the W chromosomes, they could promote a short homologous area, which would slow down the differentiation of the W chromosome and stop the turnover processes. In this context, we can assume that TauSat29 may be located in the pseudoautosomal regions (PARs) since it is present on the Z and W sex chromosomes of several species (TAL, TGU, TNE, and TSI). During male meiosis, PARs enable a proper sex chromosomal segregation [59,60]. In humans, the PAR1 region has been characterized, uncovering the presence of at least two satellite DNAs (*kalyke* and *pasiphae*) that map the telomeres of the short arms of the X and Y chromosomes [61,62]. Therefore, given that species with a common ancestor frequently retain genetic features that have been conserved over time, the similar origin of the sex chromosome system in *Triportheus* species suggests that the PAR may be a conserved region in all species

It is important to recapture that SatDNAs are very dynamic, and their evolution is mediated by molecular drive [63]. They are capable of experiencing quick changes in their nucleotide sequences, copy numbers, monomer lengths, and chromosomal positions [64,65]. Ohno’s hypothesis [1] states that sex chromosomes are descended from a pair of formerly identical autosomes that stopped recombining and therefore became distinct. Accordingly, although not detectable by FISH, our current results confirmed the Z chromosome of TAU also contains low-copy numbers of all TauSatDNAs accumulated on the W chromosome (Supplementary Figure S1). Therefore, these sequences were already present in the proto-sex ancestral ZW chromosomal pair but were nevertheless highly amplified only in the W chromosome. Additionally, from the bulk of SatDNAs analyzed, only six TausatDNAs (namely TauSat07, TauSat08, TauSat14, TauSat17, TauSat22, and TauSat29) have also accumulated in the Z and/or W chromosome of at least one other *Triportheus* species (Figure 1, Figure 2, Figure 3 and Figure 4). Therefore, as the speciation process was taking place and the W chromosomes followed their differentiation path, the majority of TauSatDNAs copies originally present on the ancestral form were deleted, most likely followed by the amplification and rise of additional species-specific SatDNAs that subsequently made up the heterochromatic portion of each of these chromosomes. Future investigations focusing on the characterization and isolation of additional Triportheus satellitomes (i.e., other than *T. auritus*) should help verify these hypotheses.

The advent of NGS technologies has opened up new possibilities for genomic comparisons of sex chromosomes throughout the evolutionary range. With numerous species having their genomes being sequenced, comparisons on a variety of issues relating to the structure, operation, and evolution of sex chromosomes had been possible [66]. However, studies aimed at discovering genes on the Y or W chromosome are highly uncommon because of the many repetitive sequences on the sex-specific chromosomes, which restrict the effectiveness of genome sequence assembly [67]. Even in groups like birds and mammals that share a conserved ZW and XY sex system, respectively, the sex-specific chromosomes are, in fact, regarded as the most variable part of the genomes and the most difficult ones to assemble. Among mammals, only the Y chromosomes of humans, chimpanzees, and Rhesus monkeys have been fully sequenced, and their structure and gene content differ substantially [68]. Accordingly, only four genes on the human Y are also located on the small marsupial Y, and there are not many shared SatDNA sequences among mammals [69]. In birds, the W chromosomes are even more variable, differing in size and gene content even among closely related species [70]. The W chromosome of snakes shows the largest heterogeneity in terms of gene content and the amplification of different repetitive sequences, even within closely related species [71]. Accordingly, our current results also point to such a rapid and divergent genomic content of the sex-specific chromosome in a short span of time (~15–25 Myr).

## 5. Conclusions

The analysis and comparison of SatDNAs in the studied *Triportheus* species helped to elucidate the dynamics of these elements in the differentiation of sex chromosomes. We demonstrated that the W female-specific chromosome in this group has undergone fast and diverse genomic evolution. In fact, the genomic composition and shape of the W chromosomes in the several studied species have undergone significant modifications throughout time. The unequal accumulation of SatDNAs on this chromosome shows that, despite their homology, *Triportheus* W chromosomes have experienced unique evolutionary processes among different species and do not follow a direct relationship of ancestry. 

## Figures and Tables

**Table 1:** *Triportheus* species investigated, with the respective hydrographic basins where they were collected, and the number of males and females sampled.

Species	Location	N
*Triportheus guentheri*	São Francisco, MG	(12 ♀; 06 ♂)
*Triportheus nematurus*	Paraguai, MT	(09 ♀; 07 ♂)
*Triportheus pantanensis*	Paraguai, MT	(05 ♀; -- ♂)
*Triportheus* aff. *rotundatus*	Paraguai, MT	(19 ♀; 21 ♂)
*Triportheus trifurcatus*	Araguaia-Tocantins, MT	(04 ♀; 11 ♂)

MG = Minas Gerais; MT = Mato Grosso Brazilian states.

## Data Availability

The data presented in this study are available on request from the corresponding author.

## Supplementary Materials

The following supporting information can be downloaded at: https://www.mdpi.com/article/10.3390/biology12081114/s1, Figure S1: Male metaphase plates of *Triportheus guentheri* (TGU), *T. nematurus* (TNE), *T. rotundatus* (TRO), and *T. trifurcatus* (TTR) highlighting the chromosomal location of TauSat07, TauSat08, TauSat17, TauSat22. and TauSat29. The SatDNA family names are indicated on the right bottom, in green (Atto488 labeled) or red (Atto550 labeled). The Z chromosomes are indicated. Bar = 5 μm; Figure S2: Positive results for the PCR products checked in a 2% agarose gel for all the 16 TauSatDNAs amplified in the microdissected Z chromosomes. TauSat01 which previously also showed FISH signals on the Z chromosome of TAU [37] was used as a positive control.
