# Supplementary figures and images for: Following the Pathway of W Chromosome Differentiation in Triportheus (Teleostei: Characiformes)
Source: Biology (Basel). 2023 Aug 10;12(8):1114. doi: 10.3390/biology12081114 (PMC10452202; doi:10.3390/biology12081114)

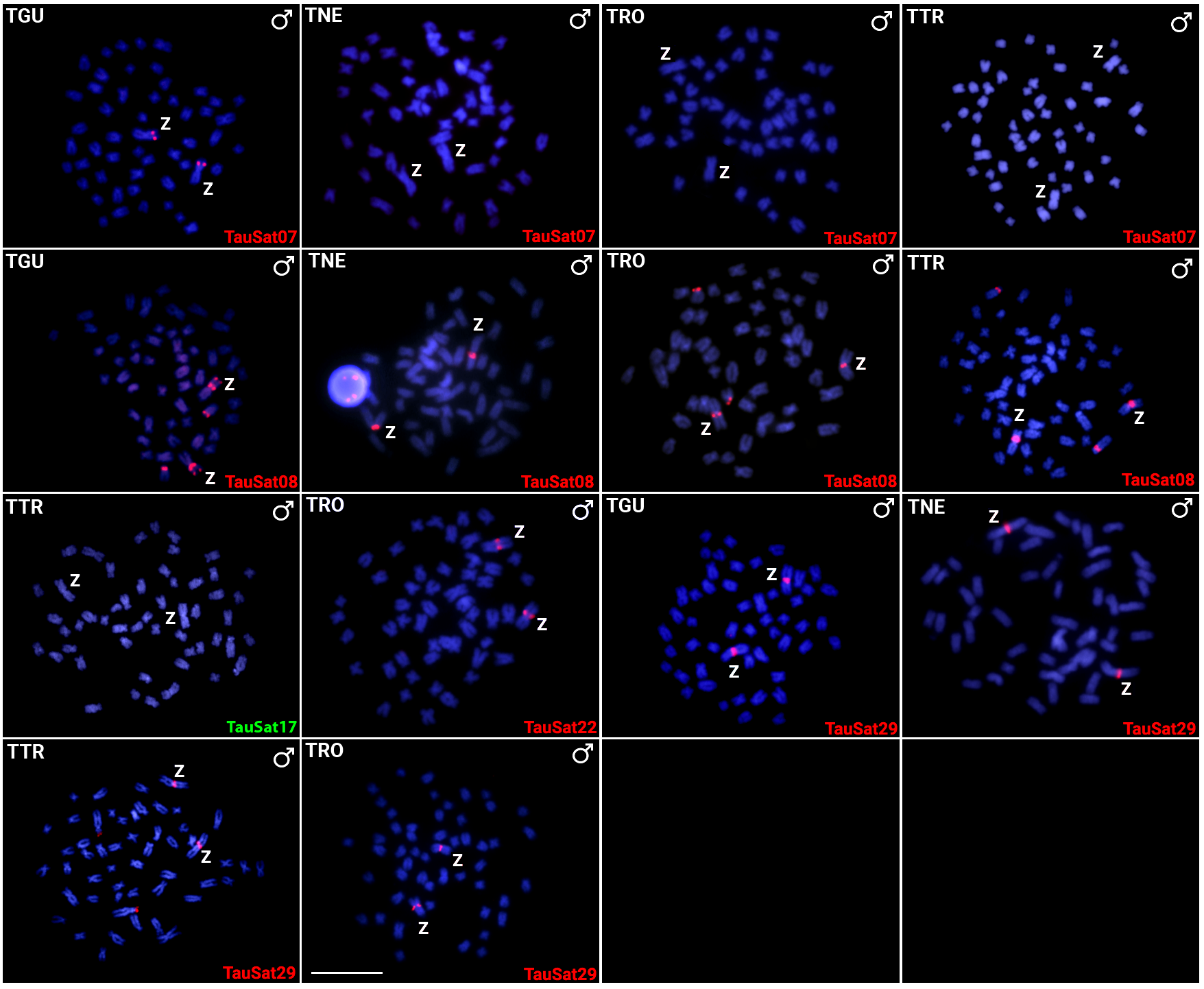

Supplement: Supplementary file 1 [file biology-12-01114-s001.zip › Figure S1.tif]

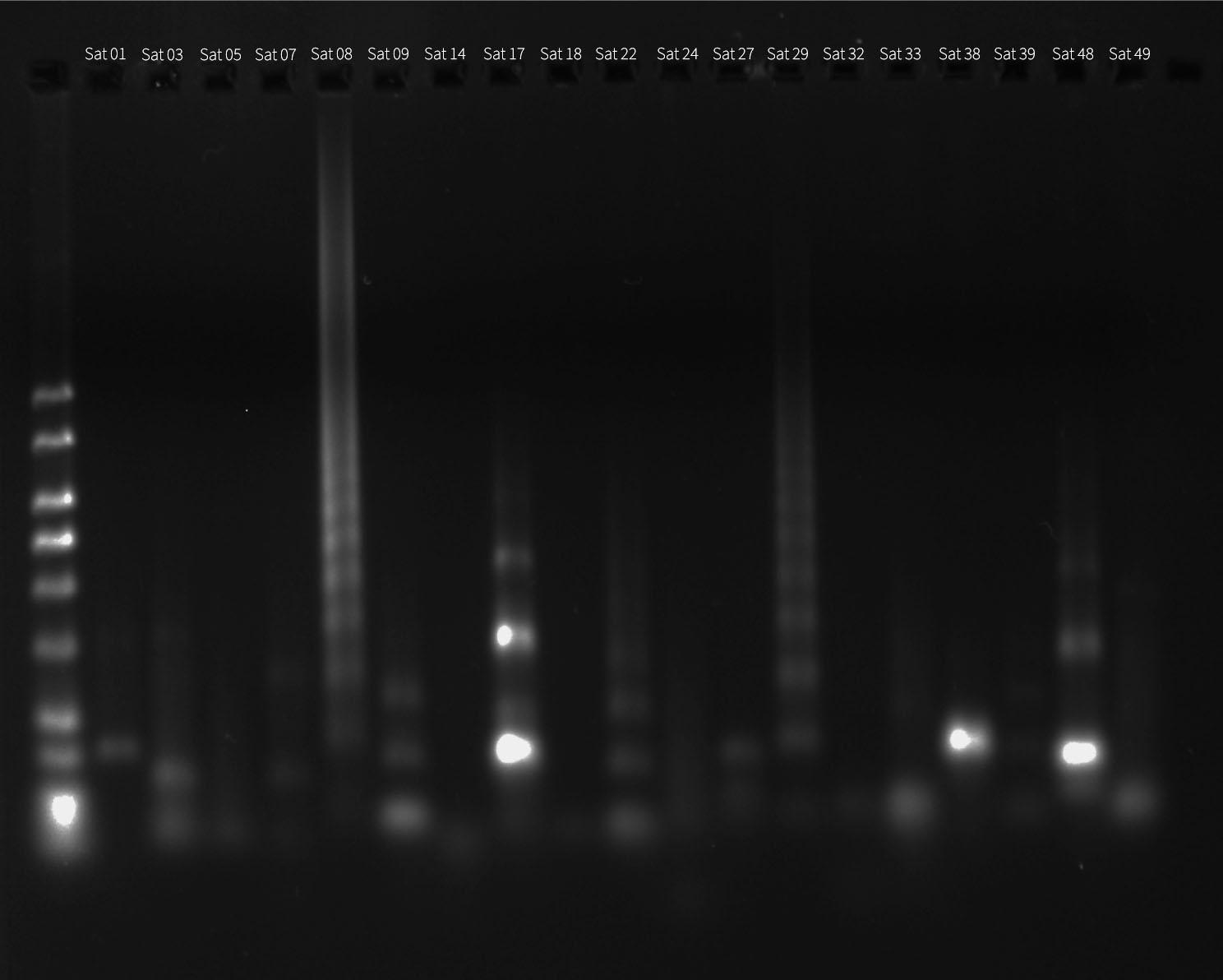

Supplement: Supplementary file 1 [file biology-12-01114-s001.zip › Figure S2.tif]
